# Cryptic etiopathological conditions of equine nervous system with special emphasis on viral diseases

**DOI:** 10.14202/vetworld.2017.1427-1438

**Published:** 2017-12-10

**Authors:** Rakesh Kumar, Rajendra D. Patil

**Affiliations:** Department of Veterinary Pathology, Dr. G.C. Negi College of Veterinary and Animal Sciences, CSK Himachal Pradesh Agricultural University, Palampur - 176 062, Himachal Pradesh, India

**Keywords:** diagnosis, encephalitis, equine, nervous system, pathology

## Abstract

The importance of horse (*Equus caballus*) to equine practitioners and researchers cannot be ignored. An unevenly distributed population of equids harbors numerous diseases, which can affect horses of any age and breed. Among these, the affections of nervous system are potent reason for death and euthanasia in equids. Many episodes associated with the emergence of equine encephalitic conditions have also pose a threat to human population as well, which signifies their pathogenic zoonotic potential. Intensification of most of the arboviruses is associated with sophisticated interaction between vectors and hosts, which supports their transmission. The alphaviruses, bunyaviruses, and flaviviruses are the major implicated groups of viruses involved with equines/humans epizootic/epidemic. In recent years, many outbreaks of deadly zoonotic diseases such as Nipah virus, Hendra virus, and Japanese encephalitis in many parts of the globe addresses their alarming significance. The equine encephalitic viruses differ in their global distribution, transmission and main vector species involved, as discussed in this article. The current review summarizes the status, pathogenesis, pathology, and impact of equine neuro-invasive conditions of viral origin. A greater understanding of these aspects might be able to provide development of advances in neuro-protective strategies in equine population.

## Introduction

The domestication of modern horses (*Equus caballus*) started approximately 5000 years ago [1]. *E. caballus* originated from same ancestor of equids around 4-4.5 years ago [2]. The equines are major contributors for providing food, transportation, employment, and income to poor farmers in many parts of the globe including India and have pivot role in sports and military activities as well. Since a century onward, this animal is continuously used in the field of science, for raising diphtheria antitoxin, antivenom sera which serves a boon for humans as well as animal lives [3,4] and recently proved as an efficient animal model for studying the human infectious conditions such as allergies [5]. Large white donkeys of Indian origin are already extinct, while most of the Indian ponies (Manipuri, Zanskari, Spiti, and Bhutia) and horse breeds (Marwari and Kathiawari) are at the verge of being declared as endangered. The most contributory factor for this decline in population of equids is the emergence of deadly diseases of equines. Among these diseases, neurological conditions play a significant role in equine death and euthanasia, as it is difficult to diagnose the origin of such encephalitic conditions [6].

The pathological conditions associated with nervous system of equines can be classified on the basis of the etiological agents involved (viral, bacterial, parasitic, fungal, algal, and plant toxins/mycotoxins) or by anatomical location of lesions, e.g., hepatic encephalopathy (hepatotoxic plants causes damage to cerebral cortex and basal nuclei) [6,7]; nigropallidal encephalomalacia mainly due to the consumption of *Rhaponticum repens* (creeping knapweed) (leads to destruction of nigrostriatal pathways causing dopamine deficiency), are the conditions mainly involving the brain [7,8] while arboviral encephalomyelitis such as Eastern equine encephalitis (EEE), Western equine encephalitis (WEE), Venezuelan equine encephalitis (VEE), and West Nile fever (WNF) [9], rabies, pelvic limb paralysis [10], and equine protozoal myeloencephalitis [11], mainly affect brain stem and part of spinal cord [6]. Equine motor neuron disease and wobbler disease (incoordination) [12], affect spinal cord, while stringhalt [13], laryngeal hemiplegia (racing thoroughbred horses) [6,14], and polyneuritis equids (caused by *Halicephalobus gingivalis*) are associated with nerves and ganglion affections. At the same time the most important diseases of horse, i.e., tetanus (sawhorse stance, spastic paralysis) and botulinum (flaccid paralysis) involve the NM (neuromuscular) junction [6], in this article, we are mainly focusing on the status and etiopathology of some of the important viruses induced neurological conditions often encountered in equines.

Alphaviruses, flaviviruses, and bunyaviruses (least important to cause encephalitis) are the major group of viruses responsible to cause encephalitis in equines (Table-1) [6,9]. The family flaviviridae contains a vast range of viruses including dengue, yellow fever, WNF, japanese encephalitis (JE), and a newly emerged virus Zika virus [15,16]. WNF and JE are of major concern as far as human and equine populations are concerned [17,18]. Horses are dead-end host for most of the arboviral diseases, except for VEE virus (VEEV) where these acts as amplifying hosts [19]. Flaviviruses are growing group of viruses which can be insect-specific, mosquitoes borne, tick-borne, and vertebrate borne in nature [20]. Within the world of RNA viruses, flaviviruses are highly pathogenic.

**Table-1:** Different groups of viruses associated with equine encephalitis along with their reservoir hosts and geographic distribution [6,9].

Different groups of viruses	Reservoirs	Geographic location	
Bunyavirus			All bunyaviruses and flaviviruses cause encephalomyelitis in general but Ross river virus in addition cause systemic hemolymphatic neurotoxic ataxia
California virus	Logomorphs and rodents	North America and part of Eastern Asia	
Alphaviruses			
Ross river virus	Marsupials and other placental mammals	Australia, Papua New Guinea	
Semliki forest virus	Unknown	East and West Africa	
EEE	Birds, rodents, snakes	North/South/Central America	
WEE	Birds, rodents, snakes	North/South America	
VEE	Cotton rat	North/Central America	
Flaviviruses			
WNF	Passerine birds	Europe, North/South/Central America, Australia, and Africa	All flaviviruses are responsible to cause encephalomyelitis. St. Louis encephalitis and Usutu are only serologically recorded
JE	Birds, swine	Russia, Asia, India, Western Pacific	
Usutu	Birds	Africa and Europe	
Murray valley	Birds, horse, cattle, foxes, marsupials	Australia, Papua New Guinea	
St. Louis encephalitis	Birds	North/South/Central America	
Kunjin virus	Waterbirds	Australia	
Powassan	Rodents, mice, skunks, dogs, birds, lagomorphs	-	
Tick-borne encephalitis	Small rodents	Asia, Europe, Russia, Finland	
Louping ill	Sheep, grouse	U.K., Iberian Peninsula	

EEE=Eastern equine encephalitis, WEE=Western equine encephalitis, VEE=Venezuelan equine encephalitis, WNF=West Nile fever, JE=Japanese encephalitis

## WNF

WNF is an emerging threat, and in the past, many outbreaks have been reported globally [21-23]. The first case of WNF was reported in Uganda in year 1937 [24] while in India 1^st^ case was reported from human in year 1952 in Mumbai [25]. This disease leads to huge mortality in horses [26] as well as in wild and domestic birds [27]. The zoonotic potential of this virus can be assessed by the cases of encephalitis in human beings [28]. The different spp. of *Culex* are the major vector species associated with the transmission of WNF virus (WNFV). In southern part of India, ardeid birds (*Ardeola grayii* and *Bubulcus ibis*) and domestic pigs (poor hosts) [29,30] have also shown antibodies against WNFV. Human and horses acts as dead-end host for WNFV. Falcon also plays an important role in the transmission of lineage 1 and 2 of WNF, which was evident by diagnostic techniques such as VNT, ELISA, and real-time reverse transcription-PCR (RT-PCR) [31]. The WNF have four lineages, namely, L1, L2, L3, and L4. Although lineages L2, L3, and L4 along with L1 were involved with many outbreaks of the world, but L1 has been found to be more significantly pathogenic [6,31]. From India L1a and L1c have been reported but L1c was found to be non-pathogenic [9]. In Italy, lineage 1 of this virus led to infection in crows, pigeons, and magpies but no severe mortalities has been noticed while great fatalities have been reported among owls, crows, and hawks in certain other parts of the world [32]. In Europe in year 2004, first time the fatalities caused by lineage 2 were reported in wild birds [33] and later on deaths among wild birds in Hungry and Austria were also reported by this lineage [34]. Human, birds, and horses also showed cases of WNF infected with lineage 2 in northern part of Greece [35-37].

Most of human cases have been reported from northeastern and southern part of India. In year 2006, a total number of 167 cases were reported and out of these 13 died. Recently an outbreak of WNF has been reported from Kerala (India) [38].

**Figure-1:**
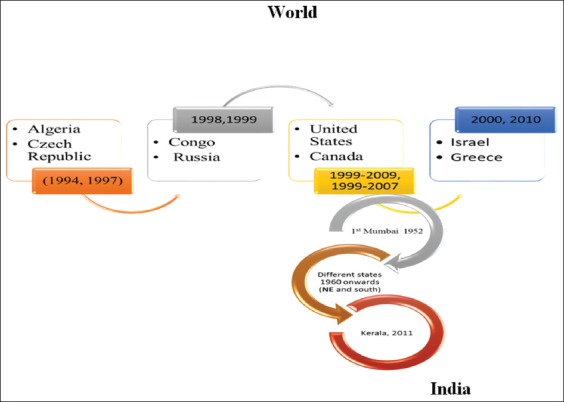
The occurrence of human cases of West Nile fever since 1994 to 2010 (upside); human cases in India stating from 1952 to 2011 (lower side). This figure is prepared by the authors with the help of SmartArt in PowerPoint.

**Figure-2:**
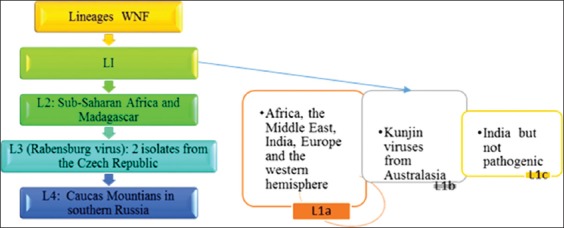
Different circulating lineages of West Nile fever virus. This figure is prepared by the authors with the help of SmartArt in PowerPoint.

WNFV from horses in India has not been documented till date, but many major outbreaks have been reported from different parts of the world. The major outbreaks have been reported from US (5674 human and 690 horse cases in year 2012) [39-41]. Outbreaks in France (in years 1962, 1965, 2000, 2003, 2004, and 2006), Italy (1998, 2008-2012), Egypt (1959), Mexico (2002, 2003), Canada (2002, 2004, 2005, 2007, 2009, and 2010), and many more parts throughout the world caused by lineage 1a (L1a) while lineage 1b reported from Australia (2011) [9]. The outbreaks caused by lineage L2 have also been reported from Hungry (2007-2011), Romania (2010) and Greece (2010, 2011). Lineages L3 and L4 were not much involved with outbreaks, but often found to be circulated among human and equine populations as shown in figures-1 and 2 [6]. The emergence of lineage L2 has been reported after 2007, as active immunization against lineage L1 of WNFV has been started in later part of year 2003 [9,23 and 39].

WNFV causes meningioencephalitis, necrotizing myocarditis, and degenerative changes in nervous system and these changes are of varying degree in different species and human beings [23,42]. The myocarditis and pancreatitis have been reported in very rare cases while non-suppurative arteritis has also been reported in falcons like raptors [43]. OAS gene of WNFV activates Ribonuclease-L, which ultimately degrade the viral nucleic acid and is responsible for pathogenic attributes of this virus [41,44]. The diagnosis of WNF can be made by seeing the color of cerebrospinal fluid (CSF), which is xanthochromic and there is a mild increase in protein values along with significant increase in mononuclear cells [9].

## JE

JE in India is important flaviviral encephalitis which is having two serotypes, i.e., JaGar-01 and Nakayama [45]. This virus is transmitted by *Culex gelidus* in all states of India, except in northern part [46] and is also transmitted by *Culex tritaeniorhynchus* through trans-ovarian route [47,48]. The *C. gelidus* along with JE have potential to transmit Ross river virus, Kunjin, and Murray valley encephalitis, as seen experimentally [49], which is an alarming indication for the spread of these deadly diseases in future. The birds are the main reservoir hosts along with pigs as major amplifying hosts, while human beings and horses are the incidental (dead end) hosts associated with this disease [6]. In southern part of India, co-infection of cysticercosis and JE in children has been reported, in which pigs were the intermediate as well as amplifying hosts for these diseases [50].

**Figure-3:**
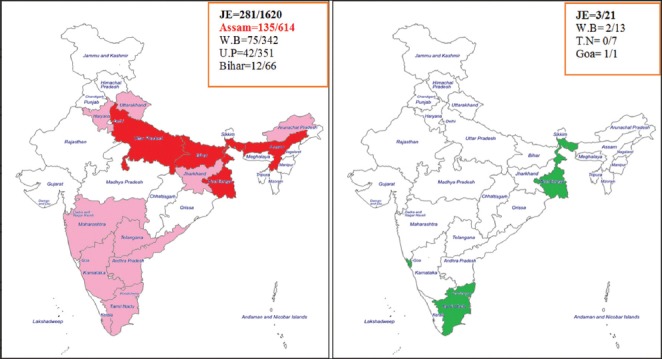
Highlighted red areas show the states with cases of Japanese encephalitis (JE) in year 2015 (left); highlighted green areas shows the states with cases of JE in year 2016 till March (right). This figure is prepared by the authors with the help of SmartArt in PowerPoint.

The first case of JE was reported in 1871 from Japan, while in India 1^st^ report has been seen in CMC Vellore in 1955 [6]. The evidence and sero-prevalence of JE among horses have been reported from Haryana (India) during 2006-2010, which were confirmed by RT-PCR. This viral strain was closely related with Vellore JE virus (JEV) isolates, i.e., H225. In year 2015, total 1620 cases of human JE were reported in India, out of these 281 people were died. The maximum number of deaths has been reported from Assam (135/614), West Bengal (74/342), Uttar Pradesh (42/351), and Bihar (12/66), shown in Figure-3. Up to March 2016, total 21 cases were reported from different parts of India, where total three people died.

In JE the blood-brain barrier (BBB) plays an important role to regulate neuro-invasion by leukocytes in late phases of infection. However, in acute stages, infiltration of inflammatory cells occurs in nervous tissue [51,52]. BBB has tight junction proteins claudin and occludin which are connected to cytoskeleton by zonula occludens (cytoplasmic proteins) and maintain the integrity of BBB [53]. However, in infections such as WNF, HIV, and JE this integrity of BBB is lost and leads to migration of inflammatory cells to parenchyma and perivascular space thereby plays a role in the development of encephalitis [54,55].

JE mainly infiltrate dendritic cells and macrophages/monocytes; thereby get access to nervous system and leads to nervous signs. CD11b+Ly-6 Chi monocytes are the characteristic hallmark cells in nervous tissue in JE, which enter into brain by breaking BBB and differentiate into macrophages, DCs and microglia cells [56-58]. In JEV infected microglia cells, Src-related Ras/Raf/ERK cascades participate in NF-KB activation and in the expression of tumor necrosis factor alpha (TNF-α) and interleukin-1 beta (IL-1β). The TNF-α and IL-1β are most important mediators for inflammatory reactions of nervous system and can be evaluated in serum and CSF [59,60]. Along with activated microglia cells, activated astrocytes are also found in JE infection in brain but microglia cells are mainly concerned with neurotoxic action [61-64].

## Henipavirus (Nipah Virus [NiV] and Hendra Virus [HeV])

**Figure-4:**
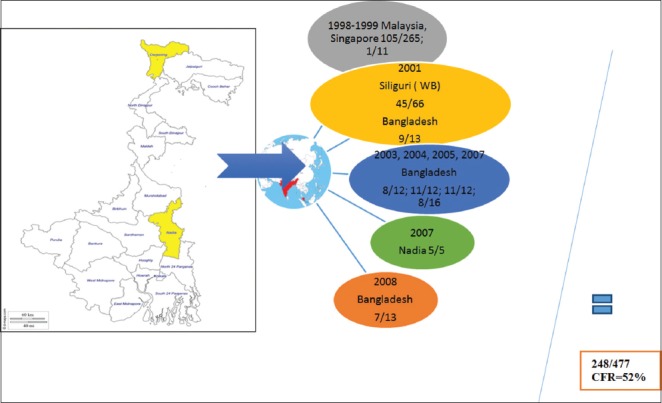
Status of Nipah virus (NiV) in India (left, yellow highlighted areas); status of NiV in the world (right). This figure is prepared by the authors with the help of SmartArt in PowerPoint.

Both NiV and HeV (-strand RNA, enveloped virus) belongs to family Paramyxoviridae and are fatal to humans as well as animals [65-68]. Fruit bats of genus *Pteropus* (*Pteropus giganteus* in Siliguri) are the major reservoir [69] for these viruses. The pigs (NiV) and horses (HeV) are the major spillover host identified. Bats besides a reservoir host often acts as a spillover host for man. Man participates as a dead end host in this transmission [70]. These viruses are zoonotic and responsible to cause threat in animals as well. However, no man-to-man transmission has been reported by NiV and HeV [71]. These viruses are responsible for many of the sporadic outbreaks of pneumonia and encephalitis in Southeast Asia [72]. First case of NiV outbreak was reported from Malaysia, isolated from the CSF of an infected patient [73]. This outbreak was a result of human and pig contacts. Later on, this virus spread to Singapore by the trading and movements of pigs [74,75]. In India, the first case of NiV has been reported in year 2001 in Siliguri (West Bengal) neighbor to Bangladesh, where already outbreaks of NiV occurred in years 2001, 2002, and 2004. The people died due to this disease until date are presented in given Figure-4. The total CFR in this entire scenario was approximately 52%, which was quite high. HeV was 1^st^ reported in Hendra, a suburb of Brisbane, Australia in 2009 which leads to the death of 13 horses due to respiratory failure [76] and later the outbreaks has been reported from Queensland between 1994 and 2008 [77]. Until date, only two human cases infected with HeV virus have been noticed, which shows meningitis [77,78]. Almost in all species, endothelial cells are the main target cells for henipaviruses [79]. NiV replicates in Dendritic cells and up-regulate the expression of CD40, CD80, and CD86 thereby affect the priming of CD8+ and CD4+ T-cells and ultimately leads to apoptosis of cells by decreasing the level of Bcl2 and elevating Caspase-3 expression. These viruses have fusion protein and glycoprotein G, and the coordinated function of these proteins helps in the attachment and entry of the virus into the host cell [80,81].

## EEE/WEE/VEE

EEV virus (EEEV)/Western virus (WEEV)/VEEV belongs to highly zoonotic new-world alphaviruses of family Togaviridae and leads to encephalitis in humans as well as in equids and are often used as potent biological weapon for bioterrorism. EEEV and WEEV lead to outbreaks to a limited area in human and horses, while VEEV causes huge epizootics in horses and spillover outbreaks in man [19,82].

**Figure-5:**
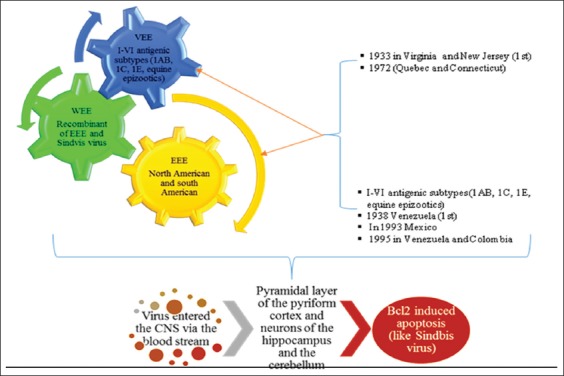
Characteristics of Eastern equine encephalitis, Western equine encephalitis and Venezuelan equine encephalitis viruses and their pathogenic mechanisms. This figure is prepared by the authors with the help of SmartArt in PowerPoint.

EEEV was 1^st^ reported from an outbreak in Vermont from Emu population, and in the same year a confirmed case of humans was also detected from the same place. EEE is mainly prevalent in northeast part of US and often involve *Culiseta melanura* and perching birds in their transmission cycle [83,84]. *C. melanura* is a potent vector as well as an occasional contributor for epidemic/epizootic transmission of EEEV. Hence, it is supposed to be a mammographic bridge vector due to its feeding habit on mammalian species (man, horse) [85-87]. Northeastern part (Massachusetts, Connecticut, Vermont, New York, and New Hemisphere) has been reported with heavy viral load of EEEV [88]. In horses, cases of EEE have been reported from Onterio (1994), Central Onterio (2001, 2002), and Nova Scotia (2009). Major outbreaks in horses have been reported in Quebec (1972), Nova Scotia (2009) and from emus in year 2008 from Quebec [6,9]. More severe form of encephalitis is caused by EEEV and WEEV than VEEV. WEEV was 1^st^ isolated from the brain of an infected horse in California in year 1930. *Culex tarsalis* is most common mosquito vector associated with this infection, and the enzootic cycle often involves a link between passerine birds and vertebral hosts (Table-2 and Figure-5) [9,89].

**Table-2:** Comparison between EEEV, WEEV, and VEEV [9,89].

Characteristics	EEEV	WEEV	VEEV (epizootic)	VEEV (enzootic)
Distribution	Eastern and Northern U.S.; South America	Western U.S.; South America	South and Central America	Southern U.S. (Florida); South and Central America
Transmission cycle	Avian-*Culiseta melanura*	Avian-*Culex tarsalis*	Unknown	Rodents-*Culex* spp.
Horse as amplifier hosts	Sometimes	No	Horses	Not known
Vector (humans/equines)	*Aedes and coquillettidia* spp.	*Culex tarsalis*	Mosquitoes	*Culex* spp.
Human mortality	50-75%	37%	1%	-
Horse mortality	70-90%	3-50%	20-80%	-
CSF examination	Color is mild turbid with increased protein content and increase infiltration of neutrophils and monocytes	Color of CSF is normal with mild increase in protein content and mononuclear cells	-	-

EEV=Eastern equine encephalitis virus, WEEV=Western equine encephalitis virus, VEEV=Venezuelan equine encephalitis virus, CSF=Cerebrospinal fluid

In 1938, Beck and Wyckoff 1^st^ time reported VEEV is a zoonotic pathogen just like EEEV and WEEV in Venezuela. *Ochlerotatus taeniorhynchus* is a major vector, and cotton spiny rat is the most common rodent host responsible for the transmission of this virus [90]. During epizootics, viremia has been developed in horses and leads to infection in mosquitoes. Hence, ultimately a spillover has been seen in human population [89,91]. EEE and WEE viruses mainly replicate in phagocytic cells, whereas VEEV virus mainly found to show neuro-invasion towards olfactory bulb [19,92,93] as has been reported in mice model with contrast to other arboviral infections. This virus mainly leads to the destruction of pyramidal layer of the cerebral cortex and hippocampal neurons by Bcl2 induced apoptosis in similar manner as shown by Sindbis virus [9] shown in Figure-5. In human, VEEV in central nervous system (CNS) leads to edema, vasculitis, and meningioencephalitis, while interstitial pneumonia in lungs, lymphocytic depletion, and degenerative changes in liver also has been reported from infected patients [19,89]. The vaccines of potent efficacy are still lacking against these viruses [94-96]. There are certain live attenuated vaccine strains like TC83 developed in past years, but these vaccines are having residual harmful effects to human beings [97]. Prophylactic as well as therapeutic protection has been reported by passive immunization with monoclonal antibodies against VEEV in mice [98,99]. DNA vaccines can also be proved as a cost-effective way for preventing viral replication [100,101].

## Equine Herpes Virus-1 (EHV-1)

EHV-1 belongs to subfamily alpha herpesvirinae, a member of genus varicellovirus and has been identified as pathogenic to domestic horses [102]. Equids act as a natural and definitive host for EHV-1. Out of nine species of EHV, only five herpes viruses (namely, EHV1, 2, 3, 4, and 5) have the ability to produce diseases in horses [103]. However, along with EHV-1, EHV-9 also has been found to be show nervous form in nature as reported in Thomson’s gazelles, which died of encephalitis in an outbreak and from calves [104]. EHV-1 and EHV-9 often found to show jumping behavior from equids to other non-equid species including polar bears, Giraffe, and Indian rhinoceros [105-107]. Both EHV-1 and EHV-2 are economically important viruses affecting the respiratory tract of horses. However, only EHV1 causes abortion and neurological disorders [102]. EHV-1 is transmitted mainly by the inhalation of the infected droplets or ingestion of EHV-1 contaminated material [108]. After entering into the body, the virus multiplication occurs in the epithelium of respiratory tract and then spread to the regional lymph nodes. Then, through leukocytes, endothelial cells of blood and lymphatic vessels spread systemically. The virus then cross the placenta, infect fetus and ultimately leads to abortion due to damage blood vessel’s endothelium.

The clinical sign includes a high fever of 102-107°F for 1-7 days, nasal discharges, coughing, abortion, and other non-specific clinical signs [109]. Abortion usually occurs after 2-12 weeks of infection in late gestation (between 7 and 11 months of gestation). In recent years, equine herpesvirus myeloencephalopathy incidences were increased, and it leads to vasculitis (due to its endotheliotropic nature), multifocal myeloencephalopathy, ischemic neuronal injury, perivascular cuffing, gliosis, hemorrhage, and thrombosis [110,111]. On rare occasions, the paralysis may advance to quadriplegia and finally death of the animal [112,113].

A number of diagnostic techniques were used for the diagnosis of EHV-1. The diagnosis of this virus by isolation, direct immunofluorescence and immunohistochemistry (IHC) is usually difficult, but PCR based assays for detection of the viral genes are quite reliable, fast and sensitive method for the detection of EHV-1 and EHV-4 [114-117]. A real-time PCR assay using allelic discrimination (E2) to distinguish between neuro-pathogenic and non-neuropathogenic strains of EHV1 has been used [118]. Similarly, the development of ORF30 region specific, allelic discrimination (E1) EHV1 real-time PCR test has also been implicated [119]. The effective vaccination together with good management practices is effective tool for prevention of EHV-1 [120]. Nowadays multivalent combined vaccines containing EHV-1, 4, EIV, EEEV, WEEV, VEEV, and tetanus are also used with promising effects against multiple infections of equines.

## Rabies

**Figure-6:**
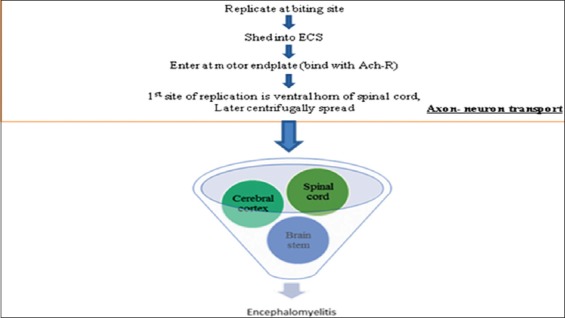
Brief overview of pathogenesis of rabies (lyssavirus). This figure is prepared by the authors with the help of SmartArt in PowerPoint.

Rabies is caused by Lyssavirus (negative strand ssRNA virus of family Rhabdoviridae). This disease often found to occur in three clinical forms including dumb, furious, and paralytic. The incubation period of this disease is approximately 15 days in experimental cases, while in clinical cases it is always fatal and leads to death within 5-10 days [6]. The behavioral changes along with progressive paralysis are the major outcomes of this disease. In contrast to other animals equines shows the involvement of spinal cord more commonly and histopathologically, most of the cases shows presence of Negri bodies in spinal cord section [10,121]. This virus is most deadly viruses in the world and having no cure if animals start showing signs and death is the final fate for the culprit. Lyssavirus mainly shows axon-neuronal transport by binding with Ach-receptors at motor end plate and multiply at the ventral horn of the spinal cord, which is the first site of replication then spread centrifugally and ultimately leads to encephalomyelitis [6]. Spinal cord is the most affected site in horse rabies cases, followed by brain stem. These portions of brain show polioencephalomyelitis along with perivascular cuffing of MNCs and formation of glial nodules on histopathological examination [10]. Fluorescent antibodies test is one of the most reliable and sensitive test, which can be used to detect the viral antigen in rabies, infected cases [6] (Figure-6).

## Diagnosis

The diagnosis of encephalitic conditions in equines is based on many factors including season, vector and host involvement, type of etiology associated, clinical, serological, and molecular results. Laboratory diagnosis of most of arboviral infections is accomplished by testing of CSF or serum samples to detect virus-specific IgM and neutralizing antibodies. Serology is the key to ante-mortem diagnosis of alphavirus and flavivirus infection. No pathognomonic signs distinguish flavivirus infection from other CNS diseases in horses. IgM capture ELISA is the test of choice to detect recent exposure to alphavirus and flavivirus. CSF from horses with acute EEEV infection typically shows a neutrophilic pleocytosis. Although WNV-infected horses can have normal CSF, if the CSF is abnormal, there is mononuclear pleocytosis. Clinically, peripheral lymphopenia can be seen in EEEV and WNV. Laboratory diagnosis of NiV can be made by virus isolation, identification of the RNA by use of RT-PCR, IHC, histopathology, or serologic tests such as indirect ELISA and virus neutralization tests. Real-time PCR on samples obtained from CSF, nasal secretions, or neural tissue can be done to detect the neuropathic strain of EHV-1. PCR testing and IHC for EEEV and EHV-1 are relatively straightforward compared with that for WNV. In recent advance researchers, pattern recognition receptors (TLRs, CLRs, RLRs, and NLRs) also found to have major role in detection of immune response against flaviviruses [44] (Table-3).

**Table-3:** Most commonly used diagnostic tests for the detection of equine encephalomyelitis causing viral diseases.

Viral diseases	Common diagnostic methods	
Diagnosis of encephalomyelitis causing viruses: • CSF examination • Cell culture isolation • Gross and microscopic examination	Detection of nucleic acids	• PCR and real-time PCR • Isothermal amplification • Restriction fragment length polymorphisms • Genome sequencing • DNA probes and DNA microarray technologies • Metagenomics
	Detection of antigens	• Ag ELISA, FAT, IHC, RIA, Immunochromatography
	Detection of antibody	• Agglutination, AGID, CFT, ELISA, bELISA, Immunoblotting
	Recent technologies	• Proteomics
		• Production of antigens by recombinant DNA technology

CSF = Cerebrospinal fluid, PCR = Polymerase chain reaction, FAT = Fluorescent antibodies test, IHC = Immunohistochemistry, RIA = Radioimmunoassay, Ag ELISA = Antigen enzyme-linked immunosorbent assay, CFT = Complement fixation test, AGID: Agar gel immunodiffusion

## Conclusion

The nervous systems affections are ubiquitous in equines worldwide and often serve as a potent source of infection to other species. Recent emergence of many diseases of equine origin has pose a threat to human civilization. The nervous system associated outbreaks (WNF, JE, NiV, EEE, WEE, and VEEE) have already been encountered globally in equines and often signifies the zoonotic potential as well. The different disease conditions associated with the nervous system shows their different spectrum of transmission and pathogenesis. Hence, nowadays, it becomes a continuous challenge to veterinarians to elucidate the different ways to establish diagnostics and therapeutic measures in more recent and advanced manner. For this, proper advances in neuroprotective strategies, proper vector control, and novel immunization strategies are highly recommended. The present review summarizes only the known viral encephalitic conditions, but still more studies are required to investigate some other unknown causes associated with the affections of the nervous system in equines.

## Authors’ Contributions

Collection, compilation and writing work along with construction of flow diagrams and tables done by RK. Editing and formatting work done by RDP. Both authors read and approved the final manuscript.
